# Updated Understanding of the Crosstalk Between Glucose/Insulin and Cholesterol Metabolism

**DOI:** 10.3389/fcvm.2022.879355

**Published:** 2022-04-29

**Authors:** Xuan Xiao, Yonghong Luo, Daoquan Peng

**Affiliations:** Department of Cardiovascular Medicine, The Second Xiangya Hospital of Central South University, Changsha, China

**Keywords:** glucose/insulin, cholesterol, statins, ezetimibe, PCSK9 inhibitors

## Abstract

Glucose and cholesterol engage in almost all human physiological activities. As the primary energy substance, glucose can be assimilated and converted into diverse essential substances, including cholesterol. Cholesterol is mainly derived from de novo biosynthesis and the intestinal absorption of diets. It is evidenced that glucose/insulin promotes cholesterol biosynthesis and uptake, which have been targeted by several drugs for lipid-lowering, e.g., bempedoic acid, statins, ezetimibe, and proprotein convertase subtilisin/kexin type 9 (PCSK9) inhibitors. Inversely, these lipid-lowering drugs may also interfere with glucose metabolism. This review would briefly summarize the mechanisms of glucose/insulin-stimulated cholesterol biosynthesis and uptake, and discuss the effect and mechanisms of lipid-lowering drugs and genetic mutations on glucose homeostasis, aiming to help better understand the intricate relationship between glucose and cholesterol metabolism.

## Introduction

**Figure 1:**
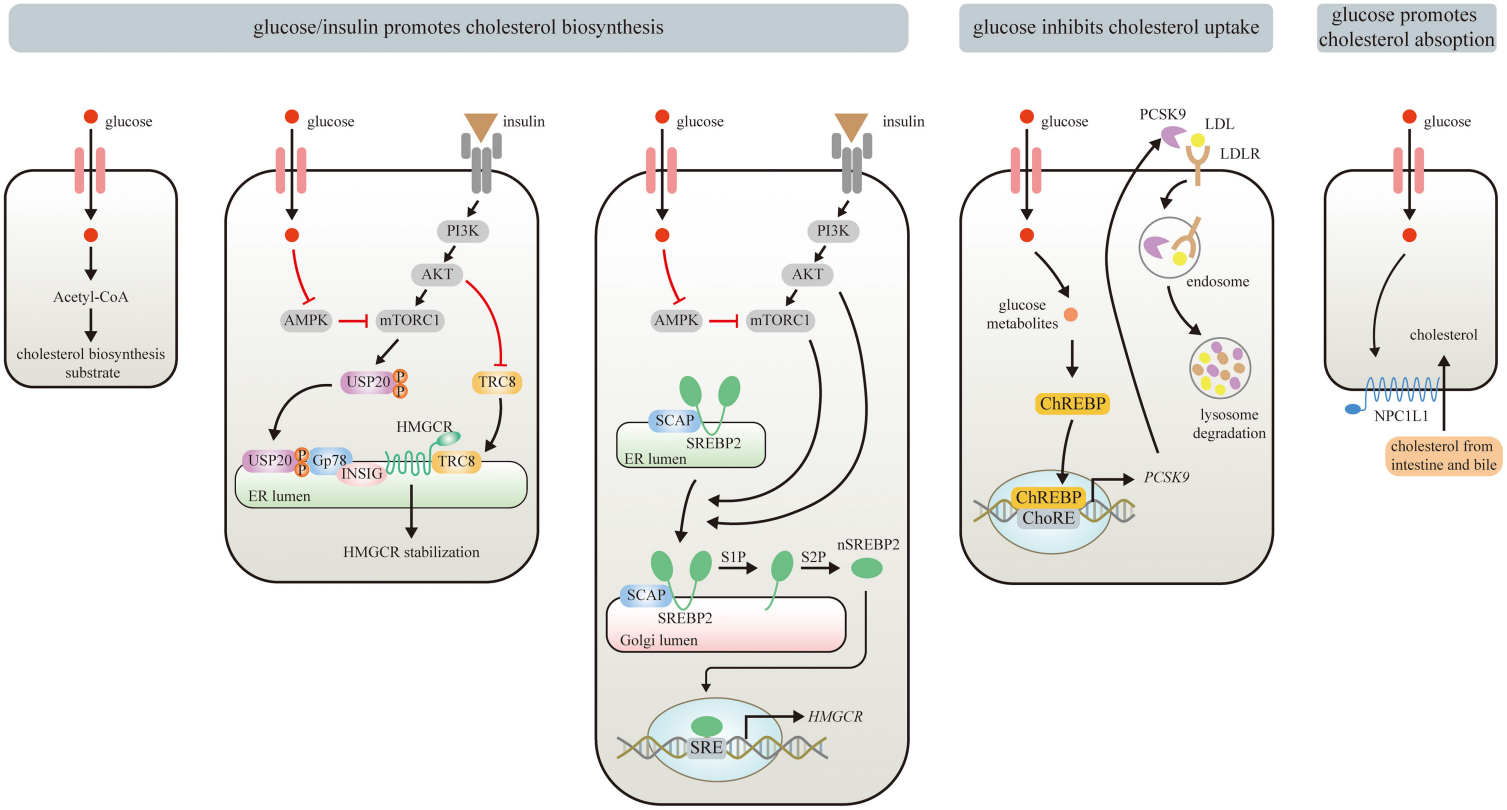
Effects of Glucose/insulin on cholesterol metabolism. Glucose provides Acetyl-CoA for cholesterol biosynthesis. Glucose/insulin also enhances cholesterol biosynthesis by stabilizing HMGCR and increasing HMGCR expression. Glucose or insulin activates mTORC1 by repressing AMPK or stimulating insulin-mediated PI3K/AKT signaling pathway, respectively. The USP20 phosphorylated by mTORC1 prevents HMGCR from being degraded by GP78. PI3K/Akt signaling pathway also stabilizes HMGCR via inhibiting the recruitment of E3 ligase TRC8. The upregulated mTORC1 can promote the translocation of SREBP2 from ER to the Golgi apparatus, where nSREBP2 is produced sequentially by the S1P and S2P. nSREBP2 translocates into the nucleus and binds to SRE sequences to stimulate HMGCR expression. Meanwhile, PI3K/Akt signaling pathway upregulates HMGCR via promoting SREBP–SCAP complex to migrate into the Golgi. Besides, glucose and its metabolites inhibit cholesterol uptake by activating ChREBP, which enters the nucleus to augment human PCSK9 expression, thereby increasing PCSK9-induced LDLR degradation. Moreover, elevated circulating glucose levels can enhance enterocyte NPC1L1 expression via some unknown mechanisms, thereby strengthening intestinal absorption of cholesterol.

**Figure 2:**
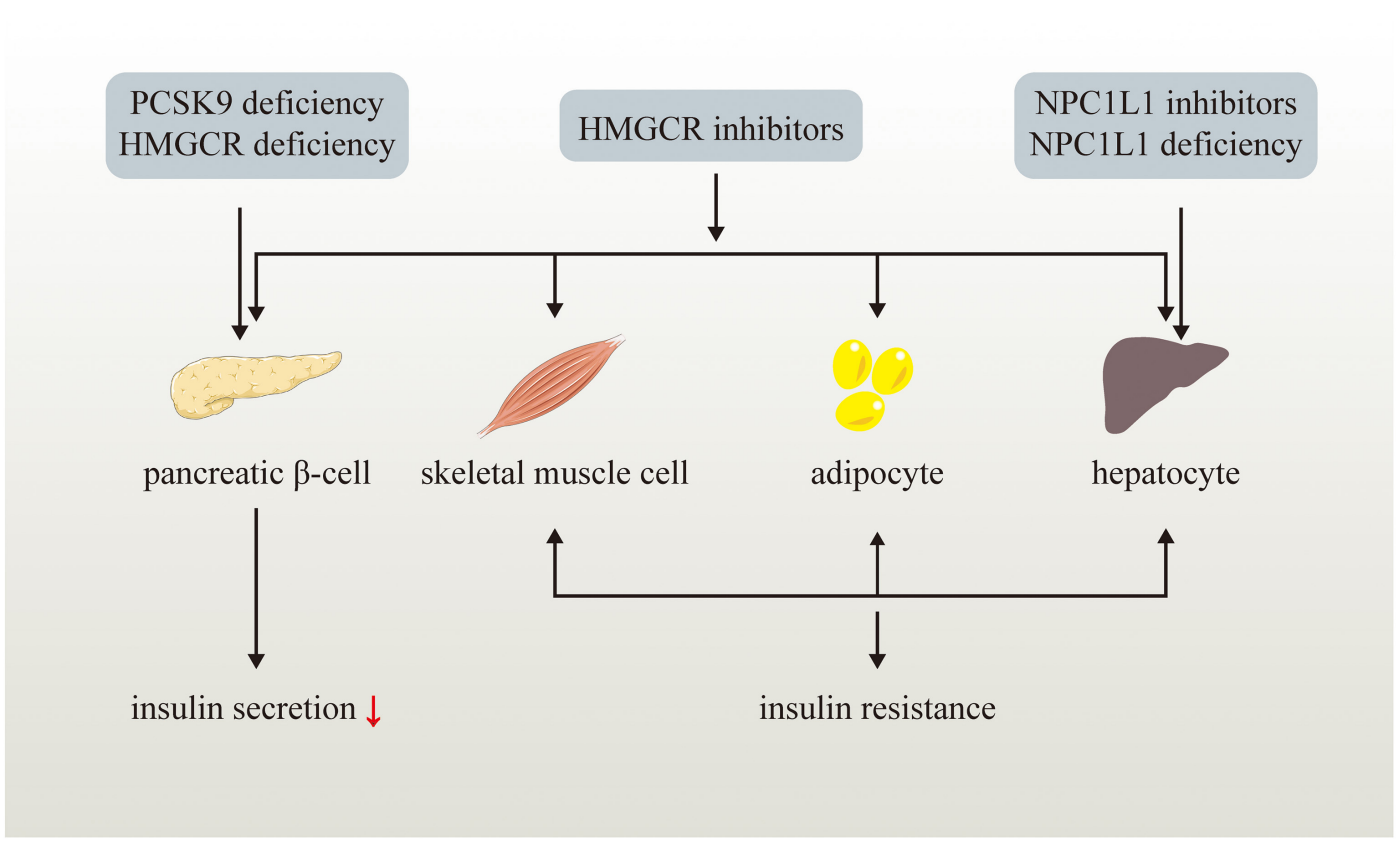
Effects of LDL-C lowering drugs or genetic variants on glucose metabolism. LDL-C lowering drugs or genetic variants disturb glucose homeostasis via multiple ways. Genetic PCSK9 deficiency impairs pancreatic β-cell insulin secretion while NPC1L1 inhibitors and genetic NPC1L1 deficiency lead to insulin resistance of hepatocyte. HMGCR inhibitors and genetic HMGCR deficiency impairs pancreatic β-cell insulin secretion and induce insulin resistance of skeletal muscle cell, adipocyte, and hepatocyte.

As indispensable nutrients, glucose and cholesterol are of prime importance in maintaining human physiological activities. In normal physiological state, hepatic gluconeogenesis and glycogenolysis maintain the normal blood glucose level for continuous consumption for energy during fasting. Glycemic hormones, including glucagon, epinephrine, glucocorticoids, and asprosin, activate a series of signal pathways of hepatic gluconeogenesis and glycogenolysis (1).Glucagon is of principal significance in endogenous glucose production among these glycemic hormones. Postprandially, the elevated blood glucose level incites insulin secretion to stimulate peripheral uptake of blood glucose, promote hepatic glycogen synthesis, and repress gluconeogenesis, thereby maintaining a normal blood glucose level (1). Since the blood insulin level increases simultaneously with blood glucose after feeding, it's difficult to distinguish the effects of insulin and glucose *in vivo*. Thus, in the following review, we may not specify the effect of glucose or insulin in some cases. However, in *in vitro* studies (e.g., hepatocytes), when using glucose as a stimulator, it is mostly the effects of glucose but not insulin. Insulin may inhibit gluconeogenesis via multiple way, such as downregulating the expression of gluconeogenesis genes, suppressing the secretion of glucagon, reducing white adipose tissue lipolysis, and cutting down skeletal muscle proteolysis (1). Generally, glucose obtained from diets, gluconeogenesis, and glycogenolysis can be decreased by experiencing aerobic oxidation for energy or converting into energy storage substances (such as glycogen and lipids) with the assistance of insulin. However, individuals with type 1 diabetes mellitus (T1DM) or type 2 diabetes mellitus (T2DM) exhibit hyperglucagonemia and hyperglycemia because of insufficient insulin secretion or insulin resistance. Besides, patients with T2DM are also characterized by defected hepatic glucose uptake and enhanced hepatic gluconeogenesis, which collectively accelerate hepatic glucose production (2, 3). Cholesterol is one of the principal lipids of cell membranes in eukaryotic cells, and the content of cholesterol influences cell membranes' physical properties and functions (1). According to current knowledge, there are two pathways for a human to acquire cholesterol: absorbing cholesterol directly from diets and synthesizing *de novo* based on acetyl-CoA, an intermediate product of glycolysis, β-oxidation of fatty acids and catabolism of amino acids (4, 5). To date, it has been proved that glucose can not only provide raw materials for cholesterol synthesis but also serve as a regulator of cholesterol biosynthesis enzymes and cholesterol uptake (as shown in Figure 1). Clinical trials indicated that the antidiabetic drug metformin reduced the blood cholesterol level in both diabetic and non-diabetic individuals (ClinicalTrials.gov Identifier: NCT01483560) (6, 7). On the other hand, cholesterol may impact glucose homeostasis, as dyslipidemic subjects treated with statin tend to develop new-onset diabetes (NOD) (8, 9). The intricate crosstalk between glucose/insulin and cholesterol has not been sufficiently discussed. This review aims to discuss the relevant roles of glucose/insulin in the biosynthesis and uptake of cholesterol based on the updated findings. Meanwhile, we will briefly summarize the relevant roles of current cholesterol-lowering drugs and cholesterol metabolism-related gene mutations in glucose regulation (as shown in Figure 2).

## Role of Glucose in Cholesterol Metabolism

### Glucose and Cholesterol Biosynthesis

#### Glucose Derived Acetyl-CoA Is the Material of Cholesterol Synthesis

Acetyl-CoA, one of the vital metabolites of glucose, serves as the direct raw material for endogenous cholesterol synthesis. Generally, acetyl-CoA deriving from glucose metabolism participates in various metabolic pathways as a substrate, such as in the tricarboxylic acid (TCA) cycle, in the acetylation reaction, and in the synthesis of ketone bodies, fatty acids, and cholesterol. The TCA cycle is initiated with the condensation of acetyl-CoA with oxaloacetate by citrate synthase, followed by the generation of citrate, the first intermediate product of the TCA cycle.

ATP-citrate lyase (ACL), which can convert citrate into oxaloacetate and acetyl-CoA, is proposed as a new target to reduce cholesterol synthesis (10). Bempedoic acid (ETC-1002) has been considered as a first-class lipid-lowering drug for its ability to inhibit the expression of ACL. Clinic trial disclosed that patients with hypercholesterolemia treated with bempedoic acid exhibit a significant reduction of low-density lipoprotein cholesterol (LDL-C) compared with placebo or standard treatment (ClinicalTrials.gov Identifier: NCT02988115) (11, 12). The beneficial effect was also observed in those who lack ample response to maximally tolerated lipid-lowering therapies (ClinicalTrials.gov Identifier: NCT02991118) (13). Meta-analyses of randomized controlled trials have shown that bempedoic acid treatment resulted in a decreased incidence of NOD (12, 14).

#### Glucose/Insulin Regulates HMGCR

It has been known since the 1970s that cholesterol biosynthesis is induced by feeding but suppressed by fasting, which is closely correlated with the activity of 3-hydroxy-3-methylglutaryl coenzyme A (HMG-CoA) reductase (HMGCR), the pivotal enzyme of cholesterol biosynthesis (15). The increased circulating glucose and insulin levels are the most remarkable changes after feeding, indicating that glucose/insulin may account for the change of HMGCR activity. It is found that the elevated glucose levels tend to downregulate the expression of adenosine monophosphate-activated protein kinase (AMPK) by lowering adenosine monophosphate (AMP)/ adenosine triphosphate (ATP) and adenosine diphosphate (ADP)/ATP ratios (16). The clinical data indicated that the first-line hypoglycemic drug, metformin, which can repress gluconeogenesis in hepatocytes via obstructing a mitochondrial redox shuttle and reduce net glucose uptake from diets by motivating anaerobic glucose metabolism of enterocytes, tended to increase AMPK and reduce serum LDL cholesterol and total cholesterol (17, 18). For example, treatment with 2.55 g/d metformin for 28 weeks reduced the plasma level of LDL-C by 14 mg/dL in 31 non-diabetic but morbidly obese individuals (7). A recent observational study including 912 participants indicated that treatment with 0.1 g/d metformin for 7 years was correlated with a 11.83 mg/dL reduction of LDL-C levels (17). Another study further revealed that plasma LDL-C levels were reduced by 16.79 mg/dL after treated with 2 g/d metformin added to titrated insulin therapy for 3 years in diabetic participants (ClinicalTrials.gov Identifier: NCT01483560) (6).

As an energy-sensing enzyme, AMPK is activated by an elevation in AMP/ATP and ADP/ATP ratios. AMPK activation tends to strengthen catabolism (e.g., glycolysis and fatty acid oxidation) but weaken anabolism (e.g., gluconeogenesis and cholesterol synthesis) (16, 19). Interestingly, it is shown that mammalian AMPK can be also restrained directly by extracellular glucose and intracellular fructose-1,6-bisphosphate in unchanged cellular energy conditions (20). The activated AMPK triggered by energy stress is likely to antagonize the biosynthetic process of cholesterol by suppressing the expression of the mammalian target of rapamycin complex 1 (mTORC1), a crucial nutrient sensor, which participates in the activation of HMGCR on the endoplasmic reticulum (ER) (21). It was reported that the repression of mTORC1 by AMPK was related to the upregulation of mTORC1 inhibitor, TSC2 gene (22). The interaction between circulating insulin and insulin receptor (INSR) phosphorylates insulin receptor substrates (IRSs), which can also enhance mTORC1 expression by initiating phosphoinositide 3-kinase (PI3K)/Akt (namely protein kinase B) signaling pathway (23). Furthermore, activation of PI3K/Akt signaling pathway caused by insulin can stabilize HMGCR via inhibiting the recruitment of E3 ligase TRC8, which can also supply an explanation for glucose/insulin-induced cholesterol synthesis (24).

Recently, Song et al. discovered that feeding would stabilize HMGCR via facilitating mTORC1 to phosphorylate Ser132 and Ser134 of the deubiquitylase ubiquitin-specific peptidase 20 (USP20), thereby protecting HMGCR from being degraded (25). Conversely, AMPK activation by fasting and metformin was likely to decrease the mTORC1 level, thereby accelerating HMGCR ubiquitination impelled by the E3 ubiquitin-protein ligase GP78 (25). Furthermore, USP20 knockout or administration of GSK2643943A, a specific USP20 inhibitor, distinctly lessened cholesterol biosynthesis after feeding compared with controls (25). Hence, it is proposed that the application of USP20 inhibitors may offer a new insight to lower cholesterol levels in hyperlipidemia (25).

#### Glucose/Insulin and SREBP2

Sterol regulatory element-binding proteins (SREBPs), a member of the membrane-bound transcription factors family, have received wide attention due to their role in regulating the synthesis of unsaturated fatty acids, cholesterol, and triglycerides (26). Three isoforms of SREBPs, namely SREBP1a, SREBP1c, and SREBP2, are encoded by SREBP1 gene and SREBP2 gene, respectively. SREBP1a and SREBP1c account for the activation of genes involved in fatty acids and triglyceride synthesis, such as fatty acid synthase (27), while SREBP2 promotes the transcription of enzymes that participated in cholesterol synthesis and uptake, including HMGCR, HMG-CoA synthase, and low-density lipoprotein receptor (LDLR) (27). When cholesterol is excess, SREBP2 is bound to SREBP cleavage-activating protein (SCAP) in ER. When the ER is deprived of cholesterol, the SREBP2-SCAP complex is transported to Golgi and SREBP2 is cleaved by two Golgi proteases (the Site-1 protease (S1P) and S2P) sequentially to release the active nuclear SREBP2 (nSREBP2). The nSREBP2 will be translocated to the nucleus and bind to nuclear sterol regulatory element (SRE) sequences, initiating the transcription of downstream genes (28).

Increased circulating glucose promotes insulin secretion after feeding. PI3K/Akt signaling pathway activated by insulin drives the movement of the SREBP–SCAP complex to the Golgi through regulating a series of classic signaling pathways, including glycogen synthase kinase-3β (GSK3β), and cyclic adenosine monophosphate (cAMP) response element-binding protein (CREB)-regulated transcription coactivator 2 (CRTC2) (27, 29, 30). Moreover, the high-glucose conditions can also enhance the stability of SCAP by directly stimulating the N-glycosylation of SCAP, facilitating the relocation of the SREBP-SCAP complex to the Golgi (31). It has been found that the mTORC1 upregulated by PI3K/Akt signaling pathway can decrease the content of cholesterol in ER by prohibiting membrane-derived cholesterol from arriving lysosomes, thereby actuating the translocation of SREBP2 from ER to the Golgi apparatus and activating cholesterol synthesis (28).

AMPK downregulation caused by increased glucose levels also prevents CRTC2 from phosphorylation, then the dephosphorylated mTORC2 is transported into the nucleus where mTORC2 enhances the transcription of gluconeogenic genes and SREBP2 (32, 33). Recently, a newly synthesized compound, Kanglexin, blockades SREBP2 signal pathway by activating AMPK, thus having the potential to lower blood cholesterol and treat atherosclerosis (34). The above evidence further elucidates the possibility to target glucose pathways to inhibit cholesterol biosynthesis and indicates that the phosphorylation of CRTC2 caused by AMPK may provide a new target to alleviate dyslipidemia, insulin resistance, and atherosclerosis.

### Glucose and Cholesterol Uptake

#### Glucose Regulates NPC1L1

The absorption of cholesterol in diets depends on Niemann-Pick type C1-like 1 (NPC1L1) protein on the apical membrane of enterocytes, which transports the cholesterol from the intestinal lumen to enterocytes (35, 36). NPC1L1 expressed in hepatocytes contributes to uptake of biliary cholesterol back to liver (37, 38). To some extent, NPC1L1 may play a role in preventing excessive excretion of cholesterol mediated by ATP-binding cassette transporter G (ABCG)5/8 heterodimer in hepatocytes and enterocytes. It has been reported that consumption of food with higher carbohydrate tends to incur higher postprandial chylomicrons (39). Several *in vitro* studies revealed that the promoter activity, mRNA levels, and protein expression of NPC1L1 in human intestinal Caco2 cells was remarkably reduced when the medium was deficient in glucose, and the promoter activity of NPC1L1 could be restored by replenishing glucose (40–42). The basolateral site of Caco-2/15 cells is responsible for sensing high glucose concentration (41), which means that the expression of enterocyte NPC1L1 may be stimulated by elevated circulating glucose levels. Unfortunately, the detailed mechanism involved in this process remains to be investigated.

#### Glucose Regulates PCSK9

PCSK9 is a plasma enzyme mainly secreted by hepatocytes but also presents in a relatively lower level in extrahepatic tissues, including the brain and the pancreas (43, 44). Recent research reveals that the presence of PCSK9 protein cannot be detected in the plasma of liver-selective PCSK9 knockout mice, indicating that the liver might be the only source of circulating PCSK9 (44). The circulating PCSK9 increases the circulating LDL-C level by promoting LDLR degradation (45). PCSK9 binds to LDLR on the plasma membrane of hepatocytes and the PCSK9-LDLR complex is then delivered to lysosomal for degradation, leading to the depletion of LDLR and subsequently elevated plasma level of LDL-C (46). Individuals with obesity and T2DM are more likely to display a higher level of PCSK9 compared with controls (47, 48). This phenomenon is probably related to the transcription of PCSK9 activated by SREBP2 due to a SRE contained in the promoter region of the PCSK9 gene (49). Interestingly, administration of metformin in patients with T2DM who had received statin treatment for more than 3 months avoided the statins-caused increase of circulating PCSK9 level in contrast with the controls without metformin treatment (50). Metformin is primarily known for inhibiting hepatic gluconeogenesis by directly restricting intracellular glucose metabolites' production (51, 52). Our latest finding reveals that the expression of the carbohydrate-responsive element-binding protein (ChREBP), a glucose sensor responsive to increased glucose and its metabolites, is repressed due to the metformin-induced reduction of intracellular glucose and its metabolites in human hepatocytes (53). ChREBP upregulates the transcription genes related to glycolysis and *de novo* lipogenesis. Previous studies have proved that the genetic deletion of ChREBP reduced levels of circulating cholesterol and LDL-C in humans and mice (54–56). We found that ChREBP activated by increased intracellular glucose and metabolites translocated to the nucleus, where it bound to carbohydrate response element (ChoRE) in the PCSK9 promoter and inducing PCSK9 transcription, eventually decreasing LDLR and elevating plasma LDL-C levels (53). Both the nuclear translocation of ChREBP and the expression of PCSK9 were notably restricted under lower intracellular glucose states triggered by metformin or glucose deprivation, but were reversed by replenishing glucose (53). Although it has been proposed that metformin may directly activate AMPK and subsequently repress fatty acid desaturase (FADS) to reduce the production of endogenous arachidonic acid, thus indirectly contributing to the recycling of LDLR via enhancing membrane fluidity (17), we observed that PCSK9 downregulation induced by metformin is unrelated to activation of AMPK and SREBP2 pathway since we did not observed changes in SREBP2 and PCSK9 expression after treated with metformin and AMPK agonists (53). The findings indicate that ChREBP has the potential to serve as a new target for hepatic PCSK9 suppression to treat dyslipidemia.

## Cholesterol-Lowering Drugs and Glucose Metabolism

### Statins

Statins are the first-line cholesterol-lowering drugs based on their ability to inhibit HMGCR (57, 58). Statins reduce intracellular cholesterol and incite SREBP2, upregulating LDLR and LDL-C uptake, thus reducing circulating LDL-C. Administration of statins in diabetic patients apparently decreased the occurrence of atherosclerotic cardiovascular disease, such as myocardial infarction (59, 60). However, increasing findings indicate that statins treatment is correlated to elevated occurrence of NOD (61, 62). Several observational studies and meta-analyses of randomized controlled trials demonstrated that statin therapy yields side effects on glucose metabolism, increasing the NOD risk by around 12% (63–66). Although the definite mechanisms behind statins-induced NOD are still uncertain, it is disclosed that statins may indirectly promote NOD by inciting pancreatic β-cells dysfunction and insulin resistance (61, 62).

#### Statins and Pancreatic β-Cells Dysfunction

Pancreatic β-cells are the only cell population that recognizes increased plasma glucose (>100 mg/dL) and secrets insulin, which is of paramount importance in controlling glucose homeostasis for its unique hypoglycemic effects, including the promotion of glycogen synthesis, glucose transport mediated by glucose transporter 4 (GLUT-4), glucose oxidation in peripheral tissues, and the suppression of glycogenolysis and gluconeogenesis.

Pancreatic β-cells dysfunction caused by statins is characterized by decreased insulin secretion. The latest findings reveal that pancreatic β-cells of HMGCR knockout mice are accompanied by severe hyperglycemia due to compromised insulin secretion and impaired pancreatic β-cell proliferation (67). The mevalonate pathway initiated by HMGCR produces isoprenoid, a kind of intermediate metabolite which contributes to insulin granule exocytosis via enhancing the posttranslational modification of small G proteins (sGPs), such as Rab5a (62). Some sGPs function as activators of mTOR, which upregulates some key pancreatic transcription factors, such as v-maf musculoaponeurotic fibrosarcoma oncogene homolog A (MafA), thereby retaining mature β-cell functional mass (68). Statins lead to isoprenoid deficiency and disturbed protein prenylation, thus impairing insulin secretion (68). Supplementation of geranylgeranyl pyrophosphate, one of the intermediates in the mevalonate pathway, significantly reverses MIN6 cells (a mouse pancreatic β-cell line) function damaged by atorvastatin (68). Hence, targeting the mevalonate pathway may also provide a new strategy for avoiding statin-induced hyperglycemia.

GLUT-2 and ion channels may also partially account for the correlation between statins and NOD. GLUT-2 transports glucose from extracellular space to cytoplasm, increasing cytosolic ATP/ADP ratio as a result of augmented glycolysis in β-cells (69). The high level of ATP is prone to stimulate instant calcium influx by blocking K^+^ -ATP channels and opening voltage-gated Ca^2+^ channel (VGCC), thereby causing exocytosis of insulin secretory vesicles (70, 71). Glucose-induced insulin secretion is decreased by repression of P2X and P2Y purinergic receptors (72, 73). It is also found that ATP and ADP present in the insulin exocytosis granules, enabling them to activate β-cell purinergic P2 receptors by serving as autocrine activators (74).

GLUT-2 expression in pancreatic β-cells is inversely proportional to the dosage of atorvastatin and pravastatin (75). Further study indicates that statins impair GLUT-2 expression, thus obstructing glucose uptake of β-cells (76). A study using MIN6 cells implied that statins deceased GLUT-2 expression by reducing the generation of ATP (77). Furthermore, it is reported that statins directly suppress VGCC expression in β-cells, eventually leading to decreased insulin secretion (78, 79).

Some findings indicate that statins promote β-cells apoptosis via prompting cytochrome c expulsion from mitochondria, providing another explanation for pancreatic β-cells deprivation and development of NOD by statins (80). Statins suppress mitochondrial complex II and III activity and reduce mitochondrial membrane potential (80, 81), which incites mitochondrial oxidative stress, eventually decreasing the synthesis of ATP and then inhibiting insulin secretion (82). A further study unveils that simvastatin may also restrain K^+^-ATP channels function directly independent of mitochondria (83).

Stains may also reduce insulin secretion by downregulating G protein-coupled receptor 40 (GPR40) and glucagon-like peptide 1 (GLP-1) receptor (84). GPR40 elevates intracellular free calcium concentration level via lessening the voltage-gated K^+^ current (85). The activated GLP-1 receptor facilitates insulin secretion by inciting adenylate cyclase, which accelerates the transformation of ATP to cAMP (86). The downstream molecules of cAMP, the cAMP-dependent protein kinase (PKA) and Epac (exchange protein activated by cyclic-AMP) 2, stimulate inositol 1,4,5-triphosphate (IP3) receptor on the ER and lead to the release of Ca^2+^ from ER, thus intensifying insulin secretion (86, 87). GPR40 and GLP-1 suppressed by statins reopen K^+^-ATP channels and decrease intracellular Ca^2+^, further hindering insulin secretion (84).

#### Statins and Insulin Resistance

The hypoglycemic effect exerted by insulin is initiated by the combination of insulin to INSR, thereby triggering the insulin signaling, including the phosphorylation of IRSs and then the activation of various kinases (such as Akt, hepatic p70 S6 kinase (S6K1), and mTOR) (88, 89). The activated Akt stimulates glycogen synthesis by repressing glycogen synthase kinase and accelerating glucose uptake via promoting GLUT-4 translocation to the plasma membrane of skeletal muscle cells and adipose tissue (9, 90). Insulin resistance, which is defined as loss of appropriate response to ordinary circulating insulin levels in insulin-targeted cells, such as hepatocytes, adipocytes, and skeletal muscle cells, is one of the pivotal causes of T2DM (91). Stains promote NOD not only by impairing pancreatic β-cells' function, but also by inducing insulin resistance.

As a vital digestive organ, the liver serves as a sensitive sensor of insulin to maintain glucose homeostasis. Insulin controls multiple hepatic metabolic pathways, such as glucose output and lipid synthesis. To date, increasing findings denote that statins therapy correlates with the aggravation of glycemic control in the liver (76). Statins stimulate hepatic gluconeogenesis by activating the key gluconeogenic genes, phosphoenolpyruvate carboxykinase 1 (PEPCK1) and glucose-6-phosphatase (G6Pase) genes (92). The pregnane X receptor (PXR) is a nuclear receptor and exert multiple functions in mediating hepatic lipid and glucose metabolism (93). Stains stimulate PXR, which prompts serum/glucocorticoid regulated kinase 2 (SGK2) dephosphorylation by the protein phosphatase 2CA (PP2CA) (92). Then, PXR and the dephosphorylated SGK2 located in the cytoplasm simultaneously transfer into the nucleus and interact with the nuclear retinoid X receptor (RXR), thereby upregulating the expression of PEPCK1 and G6Pase (92). In contrast, a different study indicates that atorvastatin increases serum glucose level by activating PXR to hamper the expression of GLUT-2 and glucokinase, rather than PXR/SGK2-mediated signaling pathway (76).

As an energy storage organ, adipose tissue also participates in statins-induced NOD as a result of the weakened insulin signal transduction process. Statins treatment is associated with decreased expression of GLUT-4 in adipocytes (94, 95). The further study indicates that statins reduce GLUT-4 translocation to the plasma membrane via inhibiting isoprenoid synthesis (95), which is indispensable for functions of Rab-4 and RhoA, two proteins facilitating GLUT-4 translocation (96). Statins also disturb the function of caveolae, where GLUT-4 inserts in the plasma membrane after being activated by insulin (97). INSR is extremely abundant in adipocyte caveolae (98, 99), which means that caveolae is required for correct insulin signaling in adipocytes. Cholesterol is essential for maintaining the characteristic shape of caveolae (100). Therefore, statins-induced cholesterol insufficiency may disrupt caveolar formation, further interrupting insulin signaling.

Skeletal muscle consumes most of the circulating glucose (~75%), and damaged glucose uptake by skeletal muscle results in T2DM (101). Therefore, statins-induced NOD may partially depend on skeletal muscle despite unclear mechanisms. Similar to adipocytes, skeletal muscle cells uptake glucose primarily via GLUT-4, and the insulin signaling may also be harmed by statins, resulting in elevated plasma glucose levels (102). It is recently found that the total expression of GLUT-4 protein in C2C12 myotubes is unaffected despite reduced GLUT-4 membrane translocation after atorvastatin treatment (103). Furthermore, simvastatin-related INSR and mTORC2 dysfunction may weaken Akt activation and disturb the phosphorylation of GSK3β in C2C12 myotubes, thus inhibiting GLUT-4 translocation (104). It is also proposed that simvastatin may incur insulin resistance in skeletal muscle by increasing fatty acid production. Simvastatin leads to acetyl CoA accumulation due to HMGCR suppression in L6 myotubes. The excess acetyl CoA acts as a precursor to enhance fatty acid synthesis, which further restrain glucose uptake by disrupting GLUT-4 translocation (105, 106). Besides interfering GLUT-4, simvastatin inhibits IR/IRS-1/Akt signaling cascade and dysregulates glycogen synthesis in skeletal muscle cells (107).

Mechanisms behind statins-induced NOD are not completely understood. Unrevealing more mechanisms may help to prevent the generation of NOD by statins.

### Ezetimibe

Ezetimibe is the only inhibitor of NPC1L1 used in the clinic to lower blood cholesterol by hindering cholesterol uptake from diet (108). Adding ezetimibe to statin therapy further reduces the plasma LDL-C both in diabetics and nondiabetics when compared with statin monotherapy (ClinicalTrials.gov Identifier: NCT00202878) (109). Long-term combination therapy with ezetimibe and acarbose improved insulin sensitivity in a high-fat diet-induced non-alcoholic fatty liver disease (NAFLD) mouse model by upregulating the mRNA expression of peroxisome proliferators-activated receptor-alpha (PPAR-α) 1 and microsomal triglyceride transfer protein (MTP) in hepatocytes (110). Besides decreasing LDL-C, ezetimibe ameliorates metabolic syndrome and reduces visceral fat (111, 112).

Interestingly, hepatic NPC1L1 overexpression inhibits hepatic gluconeogenesis and ameliorates glucose metabolism in diabetic mouse models via repressing forkhead box O 1 (FoxO1) and reducing G6Pase and PEPCK expression (113). It is reasonable to estimate that NPC1L1 suppression by ezetimibe may impair hepatic glucose metabolism and increase the risk of diabetes. However, we still lack experimental evidence to confirm the association between ezetimibe and NOD, and the underlying mechanisms remain to be investigated.

### PCSK9 Inhibitors

PCSK9 inhibitors, including anti-PCSK9 monoclonal antibodies and anti-PCSK9 vaccines (114–116), lower LDL-C by cutting down PCSK9-mediated LDLR degradation and promote hepatic LDL uptake from circulation. PCSK9 inhibitors further decrease blood cholesterol in individuals with statin tolerance (117, 118), which is partially due to elevated PCSK9 expression by statins-induced SREBP-2 activation (48, 49). Up to now, only two anti-PCSK9 monoclonal antibodies, namely evolocumab and alirocumab, have received approval on the hypercholesterolemia treatment from the United States Food and Drug Administration and the European Union (117). Numerous anti-PCSK9 vaccines are still in preclinical or clinical phases to confirm their safety and efficacy (115).

Appropriate PCSK9 expression is beneficial to maintain intracellular cholesterol homeostasis by restricting LDLR level, thereby avoiding excessive cholesterol accumulation in β-cell (44). Excess cholesterol in pancreatic β-cells undermines glucose-stimulated insulin secretion by disturbing the function of organelles, GLUT-2 and K^+^ -ATP channels, resulting in hyperglycemia (119). However, unlike statins, observational studies and meta-analyses show that alirocumab or evolocumab does not lead to NOD or aggravate preexisting diabetes mellitus (120–122). The latest findings reveal that it is the local rather than circulating PCSK9 accounts for the upregulated expression of LDLR in pancreatic β-cells, consequently incurring cholesterol overload and β-cells dysfunction (44). PCSK9 existing in pancreatic islets is derived from pancreatic δ-cell (44), which implies that the expression of PCSK9 and LDLR in the pancreas can be exempt from changes in circulating PCSK9. Both alirocumab and evolocumab primarily target liver-derived circulating PCSK9, thereby exerting finite influence on β-cells dysfunction and NOD (44). Besides, PCSK9 deficiency does not harm insulin signaling in hepatocytes and skeletal muscle cells (44). Hence, PCSK9 inhibitors may manage hypercholesterolemia without disturbing glucose metabolism.

## Cholesterol-Lowering Gene Variants and Nod

### HMG-CoA Reductatse Gene and NOD

Mendelian Randomization (MR), which utilizes genetic mutations as an instrumental variable for studying exposure factors, has emerged as a popular approach to mimic the association of exposure factors with the corresponding disease. MR approach is less susceptible to multiple confounding factors and may supply rational evidence of causation. Researchers have used this approach to explore the relationship between statins therapy and the incidence of diabetes (123, 124). A large genetic analysis based on 2,23,463 subjects showed that the amount of rs17238484-G allele, an HMGCR genetic variant used to imitate HMGCR inhibition by statins, is positively related to the degree of body weight gain and the risk of developing NOD (124). It is found that each supplementary rs17238484-G allele is correlated to a statistically significant odds ratio (OR) of 1.02 for T2DM (124). Meanwhile, genetic analysis of randomized trials including 12,9170 individuals observed a statistically significant OR 1.12 for statins-induced NOD at a mean follow-up of 4.2 years (124). Hence, the application of HMGCR gene variants proves that statin-induced HMGCR inhibition might explain the occurrence of NOD.

### NPC1L1 Gene and NOD

Similar to HMGCR alleles, LDL-C-lowering NPC1L1 alleles are also utilized as genetic alternatives to mimic ezetimibe efficacy (125). A genetic meta-analysis of 50 775 T2DM individuals and 270 269 controls observed that per genetically foreseen 1 mmol/L decrease in LDL-C by NPC1L1 variants is associated with a significant OR of 2.42 for developing T2DM (125). Although the cholesterol-lowering effect of ezetimibe has been widely accepted, the application of ezetimibe is also likely to augment the risk of T2DM based on this genetic study (125).

### PCSK9 Gene and NOD

PCSK9 genetic variants can be divided into gain of function (GOF) mutations and loss of function (LOF) mutations according to their effects on circulating LDL clearance (126, 127). Considering the interaction between LDLR and PCSK9, flow cytometry analyses detect the expression level of LDLR in HEK293 cells transfected with PCSK9 variants, which may be an effective and reliable way to distinguish these two distinct types of PCSK9 variants (128). PCSK9-GOF variants tend to increase LDLR degradation in multiple cells, followed by the high level of plasma LDL-C (129). On the contrary, PCSK9-LOF mutations are more likely to increase LDLR expression in various cells, including pancreatic β-cell, which contributes to LDL-C removal from circulation but enhances cholesterol accumulation in pancreatic β-cell. Excess cholesterol accumulation results in β-cell dysfunction, promoting the development of hypoinsulinemic hyperglycemia and impaired glucose tolerance (119). Several clinical and experimental studies unveiled that individuals with PCSK9-LOF variants tended to have higher circulating glucose levels and elevated incidence of T2DM despite lower LDL-C levels (125, 130). Familial hypercholesterolemia is mainly caused by LDLR-LOF or PCSK9-GOF. The probability of patients with familial hypercholesterolemia developing into T2DM is much lower than their unaffected relatives (131), which indirectly means that PCSK9-GOF variants might be associated with NOD. However, it should be noted that the effect of PCSK9 genetic variants on T2DM risk is different from alirocumab and evolocumab, which mainly target liver-derived circulating PCSK9 rather than systemic PCSK9.

## Conclusion

Glucose/insulin promotes cholesterol biosynthesis and cholesterol uptake, which indicates that drugs targeting lowering glucose may help to control hypercholesterolemia. On the contrary, cholesterol-lowering drugs or genetic variants could impair glucose homeostasis and lead to diabetes by decreasing pancreatic β-cell insulin secretion or inducing insulin resistance of skeletal muscle cells, adipocytes, or hepatocytes. Understanding the crosstalk between glucose metabolism and cholesterol metabolism, such as glucose-ChREBP/HMGCR- cholesterol pathway, may help to locate safe therapeutic targets for controlling both glucose and cholesterol dysregulation.

## Author Contributions

DP was the originator and supervisor of the project. DP and YL conducted elaborate polishment on the article. XX collected and analyzed relevant literature, then completed the writing of the first draft of the article. All authors read and agree with the final manuscript. All authors contributed to the article and approved the submitted version.

## Funding

This project was supported by grants from National Natural Science Foundation of China (No.81870336 to DP).

## Conflict of Interest

The authors declare that the research was conducted in the absence of any commercial or financial relationships that could be construed as a potential conflict of interest.

## Publisher's Note

All claims expressed in this article are solely those of the authors and do not necessarily represent those of their affiliated organizations, or those of the publisher, the editors and the reviewers. Any product that may be evaluated in this article, or claim that may be made by its manufacturer, is not guaranteed or endorsed by the publisher.
